# Subchronic Toxicity Study in Rats of Two New Ethyl-Carbamates with Ixodicidal Activity

**DOI:** 10.1155/2014/467105

**Published:** 2014-04-10

**Authors:** María Guadalupe Prado-Ochoa, Víctor Hugo Abrego-Reyes, Ana María Velázquez-Sánchez, Marco Antonio Muñoz-Guzmán, Patricia Ramírez-Noguera, Enrique Angeles, Fernando Alba-Hurtado

**Affiliations:** ^1^Programa de Maestría y Doctorado en Ciencias de la Producción y de la Salud Animal, Universidad Nacional Autónoma de México, 04510 Ciudad Universitaria, D.F., Mexico; ^2^Laboratorio de Química Medicinal, Departamento de Ciencias Químicas, Facultad de Estudios Superiores Cuautitlán, Universidad Nacional Autónoma de México, 54714 Cuautitlán Izcalli, Mexico; ^3^Departamento de Ciencias Biológicas, Facultad de Estudios Superiores Cuautitlán, Universidad Nacional Autónoma de México, 54714 Cuautitlán Izcalli, Mexico

## Abstract

Female and male Wistar rats were used to determine the subchronic oral toxicities of two new ethyl-carbamates with ixodicidal activities (ethyl-4-bromphenyl-carbamate and ethyl-4-chlorphenyl-carbamate). The evaluated carbamates were administered in the drinking water (12.5, 25 and 50 mg/kg/day) for 90 days. Exposure to the evaluated carbamates did not cause mortality or clinical signs and did not affect food consumption or weight gain. However, exposure to these carbamates produced alterations in water consumption, hematocrit, percentages of reticulocytes, plasma proteins, some biochemical parameters (aspartate aminotransferase, gamma-glutamyl transpeptidase, cholinesterase, and creatinine activities), thiobarbituric acid reactive substances, and the relative weight of the spleen. Histologically, slight pathological alterations were found in the liver that were consistent with the observed biochemical alterations. The nonobserved adverse effect levels (NOAELs) of the evaluated carbamates were 12.5 mg/kg/day for both the female and male rats. The low severity and reversibility of the majority of the observed alterations suggest that the evaluated carbamates have low subchronic toxicity.

## 1. Introduction

Carbamates are relatively simple molecules that are characterized by being carbamic acid esters. These molecules have been used as pesticides in agriculture, for human drug therapies (e.g., for the treatment of Alzheimer's disease, myasthenia gravis, glaucoma, and as a prophylactic for organophosphate compound poisoning), and in veterinary medicine as antiparasitic drugs [1]. The new carbamates (designed and synthesized in FES-Cuautitlan-UNAM), ethyl-4-bromophenyl-carbamate (LQM 919) and ethyl-4-chlorophenyl-carbamate (LQM 996), inhibit ovogenesis, damage ovary cells, and, therefore, affect the reproduction of the cattle tick* Rhipicephalus microplus *(effective doses LQM 919 = 0.687 mg/mL and LQM 996 = 0.279 mg/mL; [2, 3]). These effects occur in both strains that are susceptible and strains that are resistant to the commercial ixodicides that are used in Mexico [2–4]. Thus, these carbamates have been suggested as an option for tick control.

The toxicities of carbamates are highly variable; some, such as aldicarb (oral LD_50_ 0.3 to 0.9 mg/kg), carbofuran (oral LD_50_ 8 mg/kg), and carbaryl (oral LD_50_ 12.5 mg/kg), are highly toxic [5]. Others, such as propoxur (oral LD_50_ 68 to 94 mg/kg and dermal LD_50_ > 2000 mg/kg), are considered to be moderately toxic [6], and benzimidazoles are considered to have low toxicity. Albendazol has an LD_50_ of 1320 to 2400 mg/kg, and mebendazol has an oral LD_50_ of 715 to 1434 mg/kg [7, 8]. Our group has demonstrated that the carbamates LQM 919 and LQM 996 are associated with low oral hazard (LD_50_ 300–2000 mg/kg) and have low dermal acute toxicities (LD_50_ > 5000 mg/kg) according to the Globally Harmonized System (GHS) of Classification and Labeling of Chemical Substances. Oral administration of high doses causes nervous system manifestations and liver damage in rats, and dermal administration of up to 5000 mg/kg of either carbamate does not produce any clinical manifestations or liver or skin alterations [9].

Aspersion or immersion baths are the application pathways for the majority of the compounds that are used to control ticks among cattle. These methods have also been suggested for the application of these new carbamates [3]. Continuously bathing cattle causes the accumulation of the compounds in the environment and prolonged contact of the animals and humans that handle them with the carbamates. Because the carbamates LQM 919 and LQM 996 were recently synthesized, their mid- and long-term toxicity effects are unknown. Thus, the purpose of this study was to evaluate the subchronic toxicities induced by oral administration of these carbamates in rats.

## 2. Materials and Methods

### 2.1. Animals

Clinically healthy Wistar rats between 7 and 8 weeks old (41 females and 41 males) and weighing between 170 and 200 g were used. The rats were housed in polypropylene cages with same-gender companions. The environmental temperature was maintained at 22 ± 2°C, the relative humidity was maintained at 30–70% and a 12/12 hour light/dark cycle was maintained. The rats were fed with commercial feed and water* ad libitum*. This study was approved by the Internal Committee for Care of Experimental Animals of the Postgraduate Program of Animal Production and Health (UNAM, Mexico).

### 2.2. Evaluated Carbamates

The carbamates used in this study were designed and synthesized at the National Autonomous University of Mexico using a benzimidazole molecule as the structural base. The carbamates were synthesized by reacting aryl- and alkylamines with sodium hydride and benzene diethylcarbonate and were then purified using column chromatography. Next, the products were recrystallized. The compounds were structurally characterized based on their infrared spectra, hydrogen and carbon-13 nuclear magnetic resonance (purity 98%), and mass spectrometry [10]. The chemical structures, nomenclatures, molecular weights, and identification codes of the evaluated carbamates are provided in Table 1.

### 2.3. Experimental Design

The rats were randomly assigned to 1 of 9 groups. The numbers of rats and treatments for each group are shown in Table 2. The selected dosages were based on previous acute oral toxicity studies [9].

The carbamates were administered in the drinking water daily for 90 days. The concentrations of carbamates were adjusted every 7 days to account for changes in the weights of the rats and average daily water consumptions. At the end of the 90 days, the rats in groups 1, 2, 3, 4, 6, 7, and 8 were euthanized using humane procedures [11]. To observe the reversibility of any clinical signs and lesions, groups 5 and 9 (satellite groups) remained under observation for 21 additional days in the absence of carbamate exposure. After this time period, these rats were also euthanized.

After euthanasia, all rats were necropsied, and the pathological findings were recorded. The liver, kidneys, brain, and spleen were weighed, and their relative weights were calculated (i.e., the % of body weight). Samples were taken from the heart, liver, stomach, intestine, kidneys, brain, spleen, testicles, ovaries, and uterus for histopathology. Furthermore, blood and liver samples were collected for biochemical analyses.

### 2.4. Clinical Observations

The rats were observed twice daily from 7 days before the commencement of exposure until the 90-day exposure to the carbamates was completed. The satellite groups were observed for additional 21 days. The skin, mucosa, eyes, respiratory frequencies, nasal secretions, salivation, presence of tremors, convulsions, changes in activity levels, posture, gait, sensory responses to stimuli, and strange behaviors were systematically evaluated according to the table proposed by Morton and Griffiths [12]. The consumption of food and water was measured daily, and the weights of the rats were evaluated once a week.

### 2.5. Histopathological Examination

For the histopathological examinations, portions of organs were fixed in 4% paraformaldehyde and embedded in paraffin. The embedded organs were cut into 5 *μ*m thick sections, the sections were stained with hematoxylin-eosin, and then the sections were observed under an optical microscope.

### 2.6. Biochemical Tests

Plasma was obtained from the blood samples and frozen at −80°C until processing. The plasma levels of aspartate aminotransferase (AST), alanine aminotransferase (ALT),  lactate dehydrogenase (LDH), and gamma-glutamyl transferase (GGT) were measured using the methods described by Reitman and Frankel [13], and Szasz [14]. The total protein, albumin, and creatinine concentrations in the plasma were determined using the methods of Westgard and Poquette [15], Gornall [16], and Bartels et al. [17]. The aforementioned determinations were carried out using kits from BioSystems. The values obtained for albumin were subtracted from the total protein amounts to calculate the concentration of the globulins in the plasma. The relationship between albumin and globulin (A/G) was calculated by dividing the concentration of albumin by the concentration of globulin. The cholinesterase activity (CHE) was determined using Ellman's [18] colorimetric method and a kit from Wiener Lab.

### 2.7. TBARS Quantification

Samples of 0.5 cm^3^ in size were collected from the livers of all euthanized rats to estimate the oxidation-reduction states of the livers of the rats that were exposed to the carbamates. These samples were submerged in a buffer solution (PBS, pH 7.2, sodium azide 15 mM, PMSF 1 mM, Triton X-100 0.1% and EDTA 5 mM) and frozen in liquid nitrogen until they were processed. The tissues were thawed on ice, the original buffer solution was discarded, new buffer solution was added, and the samples were mechanically homogenized, sonicated (3 pulses, 10 seconds, 50% amplitude), and centrifuged (13000 g, 7° to 4°C). An equivalent volume of 2.5% perchloric acid was added to the supernatant (40 *μ*L) before incubation for 10 minutes at ambient temperature. Afterwards, the samples were centrifuged at 13000 g for 10 minutes at 4°C, and the supernatant was reacted with 0.067% thiobarbituric acid (TBA) at 90°C for 30 minutes [19]. The TBARS contents of the samples were quantified using a standard curve generated from known concentrations of malondialdehyde (MDA) at 532 nm [20]. TBARS concentrations are expressed in nmol/mg of protein.

### 2.8. Statistical Analysis

The data regarding weight gain, relative organ weights, food and water consumption, hematocrit, percentages of reticulocytes in the blood, plasma levels of AST, GGT, LDH, ALT, CHE, and creatinine, and TBARS concentrations in the liver were analyzed using a one-way ANOVA, and differences between the means were established with Fisher's* post hoc *tests (i.e., minimum significant differences) using the minimum confidence level of 95%.

## 3. Results

### 3.1. Clinical Signs

The administration of the carbamates LQM 919 or LQM 996 did not cause mortality in any rats. No clinical signs or adverse effects associated with toxicity were observed in the rats that were exposed to the carbamates throughout the 90 days of exposure or during the additional observation time of the satellite groups.

### 3.2. Food and Water Consumption

**Figure 1:**
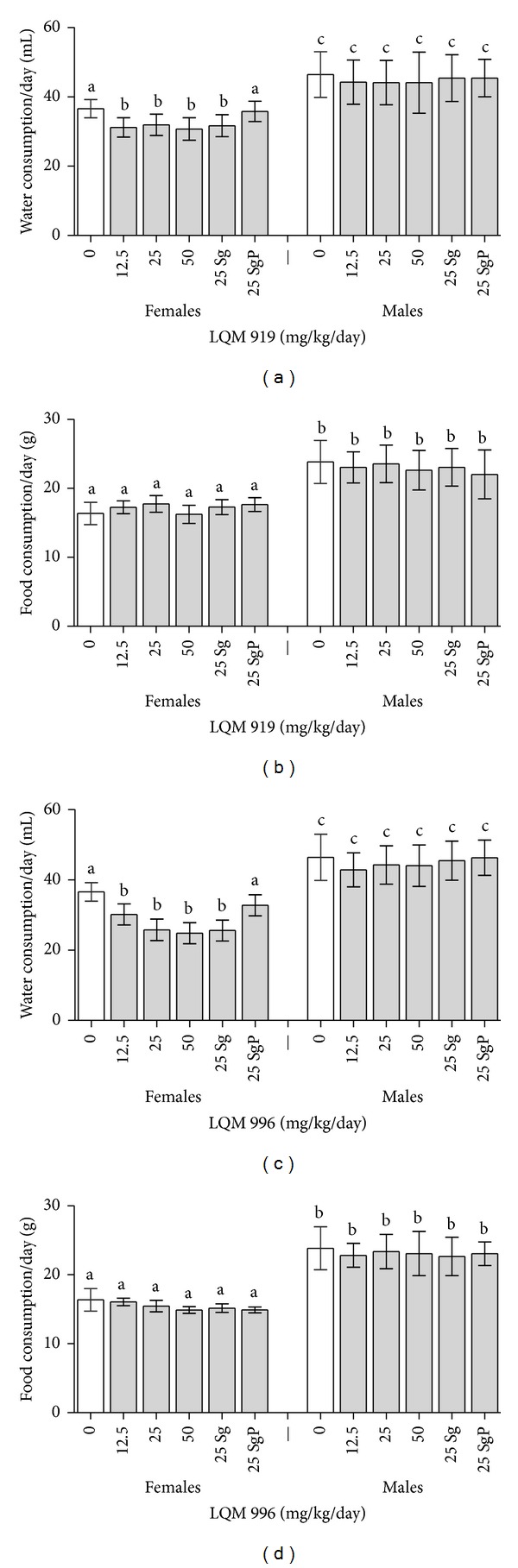
Food and water consumptions of the female and male Wistar rats that were subchronically (90 days) exposed to LQM 919 or LQM 996. Sg = satellite group during carbamate exposure (90 days), SgP = satellite group during the recovery time (21 days). Different letters indicate significant differences between the means (*P* < 0.05).

The average daily food and water consumptions are shown in Figure 1. The administration of the carbamates LQM 919 or LQM 996 did not decrease food consumption among the rats of any of the exposed groups (*P* > 0.05) compared to the rats in the unexposed groups (control groups). The females of the groups that were exposed to either carbamate, including the rats in the satellite groups, exhibited reduced (*P* < 0.05) average daily water consumption during the period of exposure compared to unexposed females. The rats in the satellite groups did not exhibit differences in daily water consumption compared to the unexposed groups in the 21 days following exposure (*P* > 0.05).

### 3.3. Body and Organ Weights

**Figure 2:**
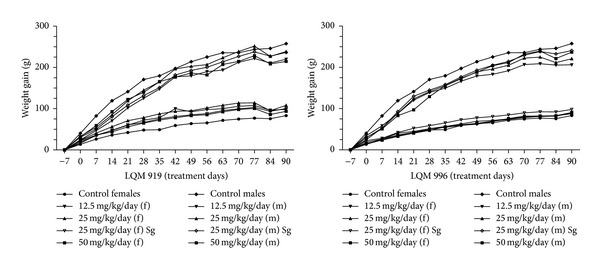
Weight gains of the female (f) and male (m) Wistar rats that were subchronically exposed to LQM 919 or LQM 996. Sg = satellite group.

The average weight gains of rats exposed to carbamates are shown in Figure 2. The administration of neither carbamate altered the weight gains of the rats of any of the experimental groups compared to the rats in the corresponding unexposed groups (*P* > 0.05).

The relative weights of the livers, kidneys, brains, and spleens of the various groups are shown in Table 3. The relative weighs of the spleen were greater (*P* < 0.05) in the female and male rats that were exposed to 25 or 50 mg/kg of LQM 919 and with all dosages of LQM 996 compared to rats of the corresponding unexposed groups. No significant differences (*P* > 0.05) were found between the relative weighs of the spleens of the rats in the satellite groups compared to the rats in the unexposed group. No significant differences (*P* > 0.05) were observed in the relative weights of the liver, kidneys, or brains of the various groups relative to the corresponding unexposed groups.

### 3.4. Biochemical and Hematological Parameters

Tables 4 and 5 show the biochemical parameters that were measured from the plasma obtained from the rats that were exposed to the evaluated carbamates. The males exposed to 50 mg/kg of LQM 919 exhibited a higher plasma concentration of creatinine and elevated total proteins and a reduced concentration of CHE compared to the unexposed males (*P* < 0.05). The females that were exposed to 50 mg/kg of LQM 919 exhibited a higher plasma concentration (*P* < 0.05) of creatinine than did the unexposed rats. The males that were exposed to 25 or 50 mg/kg of LQM 996 exhibited higher plasma concentrations of GGT (*P* < 0.05) than did the unexposed males. No significant differences (*P* > 0.05) in any of the remained evaluated plasma parameters were observed between the exposed and unexposed rat groups.

The males that were exposed to 50 mg/kg of either of the carbamates exhibited elevated hematocrit (*P* < 0.05) compared to the unexposed groups. The exposure of female and male rats to the evaluated carbamates caused an increase in circulating reticulocytes (*P* < 0.05) compared to rats in the unexposed groups. Twenty-one days after the final exposure, the satellite groups did not show any significant differences compared to the rats in the unexposed group.

### 3.5. Histopathology

**Figure 3:**
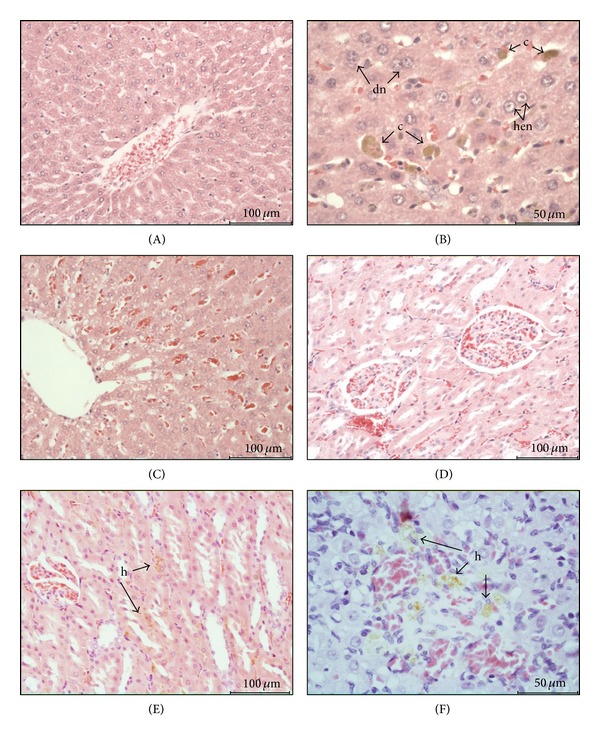
Histopathological findings from the rats that were subchronically exposed to the carbamate LQM 919. (A) Liver section (10x) of an unexposed rat (control). (B) Liver section (20x) of a rat that was exposed to 50 mg/kg/day of LQM 919 that exhibits highly euchromatic nuclei (hen), hepatocytes with two nuclei (dn), and colestasis (c). (C) Liver section (10x) that exhibits a degenerated area and congestion. (D) Kidney section (10x) of an unexposed rat (control). (E) Kidney section (10x) of a rat that was exposed to 50 mg/kg/day of carbamate that shows hemosiderin deposits (h). (F) Ovary section (20x) of an exposed rat (50 mg/kg/day) showing hemosiderin deposits (h).

The histopathological changes observed in the groups that were exposed to 25 or 50 mg/kg of either carbamate, including the satellite groups, were similar. Moderate albuminous degeneration, slight periacinar vacuolar degeneration, moderate diffuse congestion, and some hepatocytes with highly euchromatic nuclei or grayish cytoplasm were observed in the livers. The kidneys exhibited slight interstitial cell infiltration. Furthermore, hemosiderin deposits were observed in the spleen, kidneys, and ovaries (Figure 3).

### 3.6. TBARS

**Figure 4:**
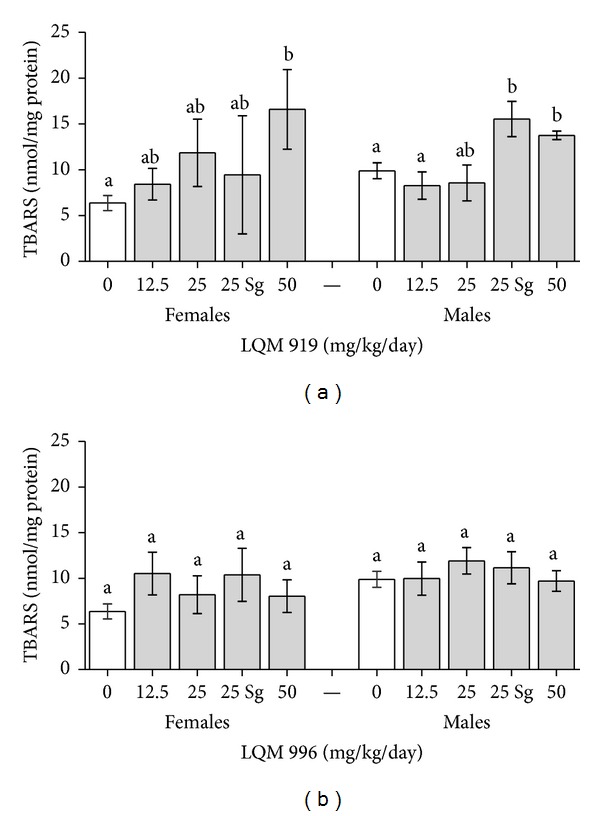
Thiobarbituric reactive substances (TBARS) in the livers of female and male Wistar rats that were subchronically exposed to the carbamates LQM 919 or LQM 996. Sg = satellite group. Different letters indicate significant differences between the means (*P* < 0.05).

Figure 4 shows the TBARS concentrations in the livers of the rats that were exposed to the evaluated carbamates. The rats exposed to LQM 996 exhibited no significant differences (*P* > 0.05) from the unexposed groups in TBARS concentrations. The TBARS levels observed after exposure to LQM 919 were significantly different from those of the unexposed group among both females and males at the maximum evaluated dosage (50 mg/kg/day). Although there were no significant dose-response effects observed in either sex, a trend was observed in the range of dosages evaluated (Figure 4).

## 4. Discussion

*In vitro* studies have demonstrated that the carbamates LQM 996 and LQM 919 inhibit the reproduction of* R. microplus* strains that are susceptible or resistant to conventional acaricides such as organophosphates, pyrethroids, and amidines [2, 3]; thus, these compounds may potentially be useful in tick control. The results of this study demonstrated that subchronic exposure to the evaluated carbamates produced slight pathological alterations in rats.

Subchronic oral toxicity studies evaluate the adverse effects caused by prolonged exposures of animals to substances and provide information about the negative effects of the substances on target organs and the cumulative effects of the substances. Furthermore, these studies determine the dosage at which no observable adverse effects are present. In this study, exposure to the carbamates LQM 919 and LQM 996 was well-tolerated, and the absence of deaths or clinical signs of toxicity related to exposure indicates that prolonged exposure to the evaluated carbamates is relatively harmless.

It has been proposed that compounds with toxic potential affect the consumption of food, metabolic processes, and, ultimately, weight gain. Studies that have evaluated the long-term effects of compounds typically consider reductions in weight gain that exceed 10% to be detrimental to the animals [21]. No differences were observed in food consumption or weight gain between the exposed and unexposed animals in this study; the weight gains of the female and male rats in the various groups all continuously increased, which suggests that there were no severe alterations of the metabolic processes of the rats that were exposed to the carbamates.

Adequate water consumption is essential for the normal physiological processes of an animal. Some substances have been observed to modify water consumption and therefore affect animals' metabolisms [22]. We found that female rats exposed to the evaluated carbamates reduced their water consumption compared to the unexposed groups. This finding may have been due to the flavor of carbamates because the females of the satellite groups regained normal water consumption levels upon termination of the exposure to carbamates. Male and female rats are known to exhibit differences in taste responses and/or taste perceptions [23], which could explain the gender differences in water consumption observed in this study. This decrease in water intake was not associated with signs of dehydration signs.

Detoxification processes primarily occur in the liver; together with the kidneys, the liver experiences the greatest exposure to xenobiotics and/or their metabolites. The susceptibilities of the liver and renal tissues to pesticide exposure-induced stress depend on the general balance between the degree of oxidative stress and antioxidant capacities [24]. Liver damage is primarily assessed via serum enzymes that include ALT, AST, LDH, and GGT. Our results showed that exposure to the evaluated carbamates caused elevations of the AST and GGT enzymes in plasma of the males, but these elevations were restricted to GGT at the maximum dosages evaluated in the females. These results, together with the minor histopathological alterations observed and the moderate increase in TBARS (which is indicative of oxidative damage), show that these carbamates are slightly hepatotoxic. Other carbamates (carbofuran and propoxur) and some organophosphates (chlorpyrifos) that are commercially used as acaricides cause similar liver alterations [25–27]. The results of the GGT analyses are interesting (Table 5) because this enzyme has been shown to have important functions in the control of cell homeostasis that are associated with oxidative stress [28]. In this study, GGT was significantly increased in the male rats that were exposed to LQM 996 at the 25 and 50 mg/kg/day dosages compared to the unexposed rats. This effect did not correlate with the TBARS estimates in which no significant differences between the exposed and unexposed animals were observed. Nevertheless, previous studies from our group have shown that same carbamate is capable of inducing significant increases in TBARS and GGT levels when administered orally to male rats at a dosage of 5 mg/kg/day in an acute exposure design [9]. Given its role as a modulator of cellular oxidative stress, specifically its function in the regulation of oxidized glutathione (i.e., the main cell antioxidant) levels [29], and its induction by subchronic exposure to LQM 919, we believe that, in the rats that were exposed to this carbamate, GGT may have exhibited redox-modulating actions (Figure 3). Therefore, if oxidative stress increases, the compensatory effects of GGT could be at risk. Based on the results obtained in this study, we suggest that further studies of the mechanisms of action of exposure to the carbamates LQM 919 and LQM 996 and their roles in the modulation of cell oxidative stress be conducted.

Susceptibility to xenobiotics can vary depending on sex, and the differences in liver susceptibilities to the evaluated carbamates between the males and females observed in this study may have been due to the greater liver metabolism and chemical breakdown capacities of the males (males possess greater quantities of cytochrome P450), which also make males more or less susceptible to the toxicity of a compound depending on whether that compound is metabolized in a bioactivation or detoxification pathway [30]. Further studies on the biotransformation of these carbamates should allow for the determination of their metabolic pathway. These results allow for the establishment of a relationship between the observations regarding the biological effects of these compounds and the mechanisms of action associated with* in vivo *and* in vitro* exposure to these compounds; this relationship is of general interest because of the potential use of these compounds as ixodicides.

Some pesticide products have been observed to have hemolytic activities. In this study, we did not directly evaluate such activity. Nevertheless, some findings, such as splenomegaly and the presence of hemosiderin in various organs (spleen, kidneys, and ovaries) suggest that the rate of erythrocyte destruction was increased. Moreover, the observed increases in reticulocytes and hematocrit in the groups exposed to the highest dosages may be related to a compensatory response of the bone marrow.

The evaluated carbamates have inhibitory effects on the development of ovary cells in* R. microplus* ticks [4], which prompted study of the effects of these carbamates on mammalian ovaries. Histological examinations of the ovaries of the rats exposed to either carbamate did not reveal any apparent pathological changes; we believe that the single deposits of hemosiderin observed in the organs of some of the rats were unrelated to any direct damage to this organ. Future studies should further evaluate the effects of these compounds on mammalian reproduction.

When a new chemical is evaluated, it is important to determine its nonobserved adverse effect level (NOAEL) to assess the risks associated with its use. Based on the results of this study, the evaluated carbamates exhibited a NOAEL of 12.5 mg/kg for female and male Wistar rats. Nevertheless, the results obtained at the 25 and 50 mg/kg dosages of the evaluated carbamates suggest caution and the need for complementary studies that evaluate the* in vivo* and* in vitro* cytotoxic and genotoxic potentials and the environmental repercussions of the carbamates LQM 919 and LQM 996.

## 5. Conclusion

The exposure to evaluated carbamates produced alterations in water consumption, hematocrit, percentages of reticulocytes, plasma proteins, some biochemical parameters, thiobarbituric acid reactive substances, the relative weight of the spleen, and slight pathological alterations in the liver. The low severity and the reversibility of these alterations suggest that the evaluated carbamates have low subchronic toxicity. The nonobserved adverse effect levels (NOAELs) of the evaluated carbamates were 12.5 mg/kg/day for both the female and male rats.

## Figures and Tables

**Table 1:** Chemical structures and molecular weights of the evaluated carbamates.

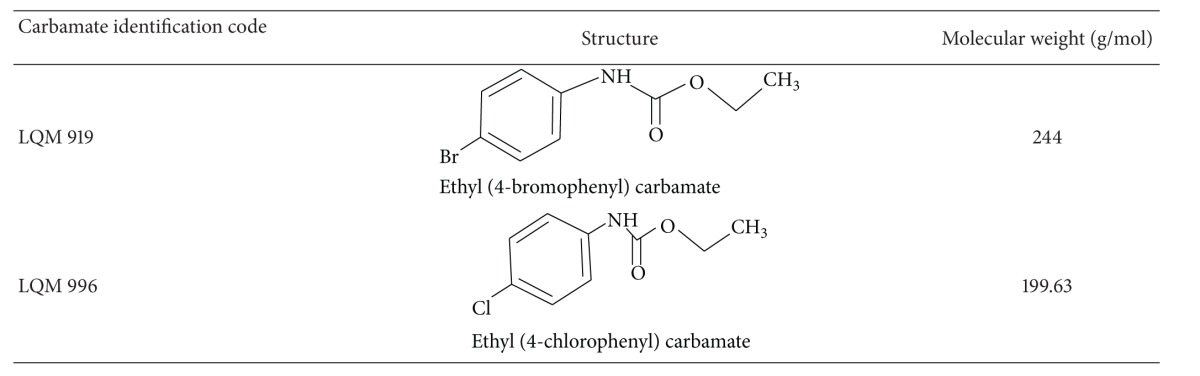

**Table 2:** Distributions of the experimental groups.

Group	Treatment per day	Rats: number and sex
1	Control group (0.1% DMSO)	5 males and 5 females
2	LQM 919 (12.5 mg/kg/day)	5 males and 5 females
3	LQM 919 (25 mg/kg/day)	5 males and 5 females
4	LQM 919 (50 mg/kg/day)	5 males and 5 females
5	LQM 919 (25 mg/kg/day), satellite group	3 males and 3 females
6	LQM 996 (12.5 mg/kg/day)	5 males and 5 females
7	LQM 996 (25 mg/kg/day)	5 males and 5 females
8	LQM 996 (50 mg/kg/day)	5 males and 5 females
9	LQM 996 (25 mg/kg/day), satellite group	3 males and 3 females

**Table 3:** Weights of the organs relative to the body weights (%) of the female and male Wistar rats that were subchronically exposed to LQM 919 or LQM 996.

Liver, kidney, brain, and spleen weights relative to body weight (%)								
Treatment	Liver		Kidney		Brain		Spleen	
	Females	Males	Females	Males	Females	Males	Females	Males
LQM 919 (50 mg/kg/day)	3.43 (±0.18)^a^	3.22 (±0.22)^a^	0.36 (±0.02)^a^	0.35 (±0.04)^a^	0.75 (±0.07)^a^	0.49 (±0.04)^a^	0.38(±0.08)^b^	0.28(±0.04)^b^
LQM 919 (25 mg/kg/day)	3.86 (±0.47)^a^	3.08 (±0.03)^a^	0.33 (±0.01)^a^	0.33 (±0.01)^a^	0.70 (±0.17)^a^	0.41 (±0.14)^a^	0.51(±0.17)^b^	0.41(±0.14)^b^
LQM 919 (12.5 mg/kg/day)	3.35 (±0.33)^a^	3.54 (±0.37)^a^	0.35 (±0.04)^a^	0.33 (±0.01)^a^	0.65 (±0.04)^a^	0.52 (±0.10)^a^	0.35(±0.04)^a^	0.22(±0.01)^a^
LQM 919 (25 mg/kg/day) Sg	3.83 (±0.01)^a^	3.60 (±0.24)^a^	0.34 (±0.04)^a^	0.34 (±0.02)^a^	0.67 (±0.05)^a^	0.38 (±0.01)^a^	0.26 (±0.00)^a^	0.18 (±0.03)^a^

LQM 996 (50 mg/kg/day)	3.96 (±0.34)^a^	3.55 (±0.24)^a^	0.33 (±0.02)^a^	0.33 (±0.01)^a^	0.71 (±0.08)^a^	0.47 (±0.04)^a^	0.47 (±0.06)^b^	0.44 (±0.08)^b^
LQM 996 (25 mg/kg/day)	3.90 (±0.53)^a^	3.60 (±0.03)^a^	0.35 (±0.02)^a^	0.35 (±0.05)^a^	0.72 (±0.09)^a^	0.48 (±0.05)^a^	0.55 (±0.07)^b^	0.39 (±0.08)^b^
LQM 996 (12.5 mg/kg/day)	3.69 (±0.39)^a^	3.46 (±0.30)^a^	0.33 (±0.03)^a^	0.33 (±0.03)^a^	0.69 (±0.10)^a^	0.50 (±0.08)^a^	0.37 (±0.04)^a^	0.31 (±0.06)^b^
LQM 996 (25 mg/kg/day) Sg	3.53 (±0.40)^a^	3.51 (±0.06)^a^	0.34 (±0.00)^a^	0.33 (±0.03)^a^	0.72 (±0.15)^a^	0.38 (±0.03)^a^	0.25 (±0.01)^a^	0.24 (±0.04)^a^

Control group	3.59 (±0.21)^a^	3.25 (±0.17)^a^	0.33 (±0.01)^a^	0.32 (±0.02)^a^	0.69 (±0.08)^a^	0.46 (±0.03)^a^	0.26 (±0.02)^a^	0.19 (±0.03)^a^

Sg: satellite group. Different letters indicate statistical differences between means (*P* < 0.05).

**Table 4:** Effects of subchronic exposure to LQM 919 on selected parameters in male and female Wistar rats.

Parameter	Group									
	Females					Males				
	Control (—)	12.5 mg/kg/day	25 mg/kg/day	25 mg/kg/day (Sg)	50 mg/kg/day	Control (—)	12.5 mg/kg/day	25 mg/kg/day	25 mg/kg/day (Sg)	50 mg/kg/day
AST (U/L)	238.2 (±78.13)	259.0 (±130.4)	152.0 (±17.12)	125.6 (±2.81)	202.3 (±72.55)	195.8 (±70.16)	166.5 (±28.12)	276.7 (±110.3)	358.2 (±49.30)*	237.1 (±62.47)
ALT (U/L)	61.52 (±19.55)	51.34 (±12.73)	68.68 (±29.46)	76.13 (±5.54)	59.20 (±9.31)	60.64 (±32.21)	76.05 (±1.96)	63.27 (±12.37)	45.97 (±10.45)	73.18 (±8.11)
LDH (U/L)	924.2 (±285.6)	851.3 (±192.9)	410.5 (±95.73)	370.7 (±62.00)	664.6 (±435.2)	739.5 (±334.1)	558.0 (±225.9)	553.7 (±246.8)	660.7 (±263.0)	872.0 (±464.4)
GGT (U/L)	1.71 (±2.28)	1.08 (±1.14)	0.77 (±0.90)	0.38 (±0.02)	0.18 (±0.26)	0.34 (±0.25)	0.51 (±0.42)	0.43 (±0.75)	0.25 (±0.42)	1.33 (±0.99)
CHE (U/L)	953.9 (±526.7)	1404 (±794.8)	982.2 (±116.6)	1132 (±444.2)	1088 (±137.8)	344.3 (±111.1)	459.0 (±138.5)	466.2 (±96.50)	344.6 (±33.32)	237.28 (±140.31)
Creatinin (mg/dL)	0.44 (±0.20)	0.64 (±0.07)	0.68 (±0.09)	0.53 (±0.16)	0.75 (±0.16)*	0.54 (±0.12)	0.65 (±0.14)	0.65 (±0.15)	0.46 (±0.04)	0.74 (±0.01)*
Total protein (g/dL)	5.83 (±1.07)	6.85 (±0.67)	7.26 (±0.43)	7.42 (±0.18)	6.61 (±0.98)	5.52 (±0.76)	6.52 (±0.29)	6.27 (±1.48)	5.80 (±0.27)	7.08 (±0.28)*
Albumin (g/dL)	2.36 (±1.05)	1.98 (±0.87)	3.71 (±0.32)	2.32 (±1.44)	3.09 (±0.77)	1.72 (±0.86)	2.72 (±0.95)	1.87 (±0.10)	2.36 (±0.28)	1.99 (±0.91)
Globulin (g/dL)	3.47 (±0.89)	4.87 (±1.45)	3.55 (±0.13)	5.09 (±1.51)	3.52 (±1.35)	3.80 (±0.27)	3.81 (±1.67)	4.40 (±1.56)	3.45 (±0.55)	5.09 (±0.82)
Reticulocytes (%)	4.21 (±0.35)	6.13 (±0.74)	5.78 (±0.72)	3.87 (±0.79)	6.66 (±1.49)*	3.93 (±0.77)	5.44 (±1.07)*	6.15 (±0.53)*	2.75 (±0.95)	5.87 (±0.66)*
Hematocrit (%)	36.6 (±2.92)	41.70 (±2.16)	33.20 (±8.78)	29.33 (±9.45)	39.80 (±6.41)	35.70 (±3.89)	43.00 (±2.82)	28.33 (±6.65)	37.33 (±3.05)	48.00 (±5.56)*
A/G	0.74 (±0.39)	0.49 (±0.45)	1.04 (±0.06)	0.54 (±0.44)	1.00 (±0.39)	0.46 (±0.24)	0.76 (±0.34)	0.48 (±0.24)	0.70 (±0.19)	0.43 (±0.29)

AST: aspartate aminotransferase, ALT: alanine aminotransferase, LDH: lactate dehydrogenase, GGT: gamma-glutamyltransferase, CHE: cholinesterase, A/G: albumin and globulin, Sg: satellite group. Values are presented as the means ± the SDs. *P < 0.05 (significantly different from the unexposed group).

**Table 5:** Effects of subchronic exposure to LQM 996 on selected parameters in male and female Wistar rats.

Parameter	Group									
	Females					Males				
	Control (—)	12.5 mg/kg/day	25 mg/kg/day	25 mg/kg/day (Sg)	50 mg/kg/day	Control (—)	12.5 mg/kg/day	25 mg/kg/day	25 mg/kg/day (Sg)	50 mg/kg/day
AST (U/L)	238.2 (±78.13)	263.4 (±82.12)	192.3 (±84.08)	181.8 (±23.10)	207.5 (±96.51)	195.8 (±70.16)	265.6 (±26.8)	167.8 (±30.81)	196.9 (±60.23)	168.3 (±18.03)
ALT (U/L)	61.52 (±19.55)	90.14 (±84.82)	44.37 (±21.49)	83.43 (±17.10)	52.26 (±13.00)	60.64 (±32.21)	74.84 (±58.61)	38.33 (±12.79)	72.70 (±15.67)	63.04 (±15.46)
LDH (U/L)	924.2 (±285.6)	908.8 (±279.8)	506.0 (±241.0)	547.0 (±74.75)	609.0 (±249.0)	739.5 (±334.1)	942.4 (±310.5)	441.0 (±146.5)	694.0 (±387.2)	607.8 (±143.7)
GGT (U/L)	1.71 (±2.28)	—	1.29 (±1.99)	1.56 (±0.36)	1.62 (±1.57)	0.34 (±0.25)	2.24 (±3.14)	6.29 (±4.82)*	2.10 (±1.58)	6.59 (±5.35)*
CHE (U/L)	953.9 (±526.7)	1505 (±348.6)	984.6 (±210.9)	1754 (±838.4)	892.8 (±317.8)	344.3 (±111.1)	546.4 (±308.4)	385.0 (±71.40)	716.5 (±488.1)	528.0 (±94.73)
Creatinin (mg/dL)	0.44 (±0.20)	0.43 (±0.07)	0.44 (±0.02)	0.38 (±0.05)	0.49 (±0.10)	0.54 (±0.12)	0.50 (±0.12)	0.60 (±0.10)	0.52 (±0.09)	0.54 (±0.17)
Total protein (g/dL)	5.83 (±1.07)	6.92 (±1.23)	5.88 (±1.12)	6.98 (±0.06)	5.93 (±0.08)	5.52 (±0.76)	5.96 (±0.39)	5.95 (±0.37)	6.26 (±0.71)	6.38 (±0.72)
Albumin (g/dL)	2.36 (±1.05)	2.40 (±1.31)	1.83 (±1.51)	2.04 (±1.09)	1.87 (±1.04)	1.72 (±0.86)	2.18 (±0.72)	2.16 (±0.92)	2.40 (±0.20)	2.01 (±0.81)
Globulin (g/dL)	3.47 (±0.89)	4.52 (±1.53)	4.05 (±1.36)	4.94 (±1.12)	4.06 (±1.01)	3.80 (±0.27)	3.79 (±0.84)	3.79 (±0.55)	3.86 (±0.84)	4.38 (±1.43)
Reticulocytes (%)	4.21 (±0.35)	8.82 (±1.61)*	10.06 (±2.15)*	2.34 (±0.25)	9.92 (±3.42)*	3.93 (±0.77)	5.23 (±1.44)*	6.65 (±1.15)*	3.38 (±0.37)	6.60 (±0.97)*
Hematocrit (%)	36.62 (±2.92)	38.00 (±6.97)	34.4 (±9.52)	35.00 (±11.00)	36.80 (±8.49)	35.70 (±3.89)	44.25 (±3.20)*	37.30 (±6.41)	41.33 (±2.51)	43.60 (±4.72)*
A/G	0.74 (±0.39)	0.64 (±0.46)	0.56 (±0.63)	0.47 (±0.34)	0.53 (±0.37)	0.46 (±0.24)	0.62 (±0.31)	0.60 (±0.30)	0.65 (±0.21)	0.55 (±0.36)

AST: aspartate aminotransferase, ALT: alanine aminotransferase, LDH: lactate dehydrogenase, GGT: gamma-glutamyltransferase, CHE: cholinesterase, A/G: albumin and globulin, Sg: satellite group. Values are presented as the means ± the SDs. *P < 0.05 (significantly different from the unexposed group).
